# Establishing the Feasibility and Acceptability of a Caregiver Targeted Intervention to Improve Pain Assessment Among Persons With Dementia

**DOI:** 10.1093/geroni/igad074

**Published:** 2023-07-13

**Authors:** Catherine Riffin, Lilla Brody, Priya Mukhi, Keela Herr, Karl Pillemer, Madeline Rogers, Charles R Henderson, M Cary Reid

**Affiliations:** Department of Medicine, Weill Cornell Medicine, New York, New York, USA; Department of Medicine, Weill Cornell Medicine, New York, New York, USA; College of Human Ecology, Cornell University, Ithaca, New York, USA; College of Nursing, University of Iowa, Iowa City, Iowa, USA; Department of Medicine, Weill Cornell Medicine, New York, New York, USA; College of Human Ecology, Cornell University, Ithaca, New York, USA; Department of Medicine, Weill Cornell Medicine, New York, New York, USA; College of Human Ecology, Cornell University, Ithaca, New York, USA; Department of Medicine, Weill Cornell Medicine, New York, New York, USA

**Keywords:** Dementia, Family caregiving, Pain

## Abstract

**Background and Objectives:**

Despite its prevalence and impact, pain is underdetected and undermanaged in persons with dementia. Family caregivers are well positioned to detect pain and facilitate its management in their care recipients, but they lack training in symptom recognition and communication. This study reports findings from a pilot trial evaluating the Pain Identification and Communication Toolkit (PICT), a multicomponent intervention that provides training in observational pain assessment and coaching in pain communication techniques.

**Research Design and Methods:**

Family caregivers of persons with comorbid pain and moderate-to-advanced dementia were randomly assigned to PICT (*n* = 19) or a control condition (*n* = 15). Caregivers in the PICT group participated in four weekly sessions delivered by telephone with a trained interventionist; caregivers in the control group received an information pamphlet about pain and dementia. All participants completed surveys at baseline and 12 weeks. Caregivers in the intervention group also completed semistructured interviews at 12 weeks. Quantitative data were analyzed using descriptive statistics and *t* tests; qualitative data were analyzed using content analysis.

**Results:**

All participants (100%) in the PICT group completed the intervention and most completed the 12-week assessment (94%). PICT randomized caregivers reported that the intervention helped them to feel more confident in their ability to recognize (67%) and communicate about pain symptoms (83%). At 12 weeks, caregivers in the PICT group showed a statistically significant improvement in self-efficacy in pain-related communication. In qualitative interviews, caregivers emphasized the utility of PICT’s components, including pain assessment tools, and offered considerations for future enhancements, such as technology-based adaptations and integration within care delivery systems.

**Discussion and Implications:**

This pilot trial demonstrates that PICT is feasible to implement, acceptable to caregivers, and has the potential to improve confidence in recognizing and communicating about pain. Results support conducting a fully powered efficacy trial, an important step toward future integration into real-world care delivery.

**Clinical Trial Registration Number:**

NCT03853291

**Translational Significance:** Persistent pain is severely underdetected and undermanaged in persons with dementia. Training dementia caregivers in observational pain assessment and subsequent communication of the assessment results to a health care provider has the potential to help address underdetection and undermanagement of pain in this population. The present research takes an important step toward achieving this objective by pilot testing a theory-driven, multicomponent intervention that is poised for future implementation and evaluation in real-world care delivery settings.

Pain is common in persons with dementia. In the United States, more than half of afflicted individuals living in community-dwelling experience daily pain and pain-related activity limitations (Atee et al., 2021; Bell et al., 2022). Pain is strongly correlated with advanced age and negatively affects persons with dementia at any disease stage or subtype (e.g., Alzheimer’s disease, vascular dementia, and frontotemporal dementia; Blytt et al., 2018; van der Leeuw et al., 2018). The causes of pain in persons with dementia are analogous to those in adults without cognitive impairment, most often originating from musculoskeletal (e.g., arthritis), gastrointestinal (e.g., peptic ulcers), and cardiac conditions (e.g., ischemia and myocardial infarction), genitourinary infections, injuries and wounds (e.g., burns and pressure ulcers; Corbett et al., 2012).

Despite its prevalence, pain is often inadequately managed in persons with dementia (Husebo et al., 2021; Moschinski et al., 2017). Data from clinical studies indicate that afflicted individuals receive fewer pain medications, including nonsteroidal antiinflammatory drugs and other classes of analgesics (opioids and acetaminophen) than persons without cognitive impairment (Achterberg et al., 2021). The negative effects of undertreated pain are significant. Suboptimal pain management not only has negative consequences for individuals’ quality of life and functional independence (Burks et al., 2021; van Dalen-Kok et al., 2021) but also can exacerbate the behavioral and psychiatric symptoms of dementia (e.g., agitation and aggression; Atee et al., 2021). Such issues may lead to inappropriate prescriptions of antipsychotic medications (Lornstad et al., 2019) and increased health service among afflicted individuals (Benner et al., 2018; Jones et al., 2022), and feelings of stress and burden among caregiving relatives (Jensen-Dahm et al., 2020; Regier et al., 2021; Smith, 2017).

Accurate pain identification is a prerequisite for effective pain management. In persons without cognitive impairment, self-report of pain remains the gold standard (Kang & Demiris, 2018). In persons with dementia, valid self-report from the patient is often complicated by problems with verbal communication and aphasia (Bullock et al., 2019; Scherder et al., 2009). In the early stages of dementia, individuals may not remember the occurrence or timing of prior pain, which is necessary for identifying its etiology and severity. Even modest changes in language processing and recall can limit individuals’ ability to describe their pain symptoms and need for treatment (Malara et al., 2016). At moderate stages, communication may be compromised due to decrements in cognitive and linguistic capacities. At advanced stages, the behavioral and psychological symptoms of dementia (e.g., agitation, aggression, and confusion) can obscure key indicators of pain (e.g., facial grimacing, moaning, and crying). In cases where self-reports of pain are not reliable, behavioral assessment is recommended (Herr et al., 2019).

## The Role of Family Caregivers in Pain Assessment and Management

The vast majority (90%) of community-dwelling persons with dementia receive assistance from family caregivers (Chi et al., 2019). These caregivers are well positioned to conduct behavioral pain assessments given their long-term relationships with their care recipient and involvement in pain management regimens, such as administering medications, supporting physical therapy regimens, and assisting with mobility (Riffin et al., 2017). Their participation in these activities affords varied opportunities to identify the presence of pain (e.g., when the care recipient is walking, bending over, or sitting) and to monitor fluctuations in pain severity over time. Caregivers are also frequent companions to their care recipients’ doctor’s appointments, and thus, have regular opportunities to communicate their observations to health care providers (Welch et al., 2022). This is particularly important as providers often rely on caregivers for information regarding the patient’s medical history, changes in cognitive and physical function, and persistent symptoms and behaviors (Riffin et al., 2021; Wolff et al., 2020).

### Caregiver Recognition and Communication About Pain in Persons With Dementia

Although caregivers can play an important role in promoting accurate pain identification and management in persons with dementia through routine assessment, monitoring, and reporting, they experience significant challenges in these areas (Bullock et al., 2019; Lee et al., 2019). Data from quantitative studies indicate that family caregivers can be poor judges of their care recipients’ pain—either under- or overestimating its presence (Amspoker et al., 2021; Hadjistavropoulos & Gallant, 2018). Qualitative work further suggests that caregivers’ uncertainty differentiating between pain and dementia symptoms can hinder their ability to serve as accurate reporters to health care providers (Riffin, Patrick, et al., 2022). Many caregivers, particularly those from racial and ethnic minority groups, are reticent to raise pain-related concerns with medical professionals (Bonds Johnson et al., 2022; Chi & Demiris, 2017) and express frustration with pain-related communication. Overall, caregivers report both a lack of knowledge regarding pain management and limited confidence in their ability to respond effectively to their care recipient’s pain (Bullock et al., 2020; Chi et al., 2022; Havyer et al., 2017).

### Interventions to Support Caregivers in Pain Recognition and Communication

More than 500 interventions have been designed to improve caregiver skills, knowledge, and coping (Butler et al., 2021; Cheng & Zhang, 2020), yet there remains a relative paucity of programs designed to help caregivers of persons with dementia with pain symptom recognition and communication. According to a recent systematic review of interventions to support family caregivers in pain management, only one was designed for caregivers of persons with dementia (Chi et al., 2020). This intervention aimed to help caregivers prevent aggressive behaviors in Veterans with comorbid pain and dementia (Kunik et al., 2017). Further, although several programs have been designed to promote cooperative communication among family caregivers and medical professionals (Pillemer et al., 2003), such interventions have been developed and tested in residential care (i.e., nursing homes) rather than community settings, where the vast majority of persons with dementia receive their care. None have focused on pain-related communication in the context of dementia (Woo, 2021).

### The Present Study

The Pain Identification and Communication Toolkit (PICT) was developed by a multidisciplinary team of experts with backgrounds in geriatric pain assessment and dementia caregiving to address the challenges that caregivers of persons with dementia face in recognizing and communicating about pain. PICT is a theory-driven, multicomponent intervention that provides (a) training in observational pain assessment, (b) coaching in effective pain communication, and (c) structured opportunities for skill-building. This article describes the development and design of PICT and reports preliminary findings from a pilot randomized controlled trial (RCT) examining PICT’s feasibility and acceptability—including its specific components—and potential to improve caregivers’ confidence in both pain recognition and communication.

## Research Design and Methods

### Theoretical Framework

**Figure 1.**
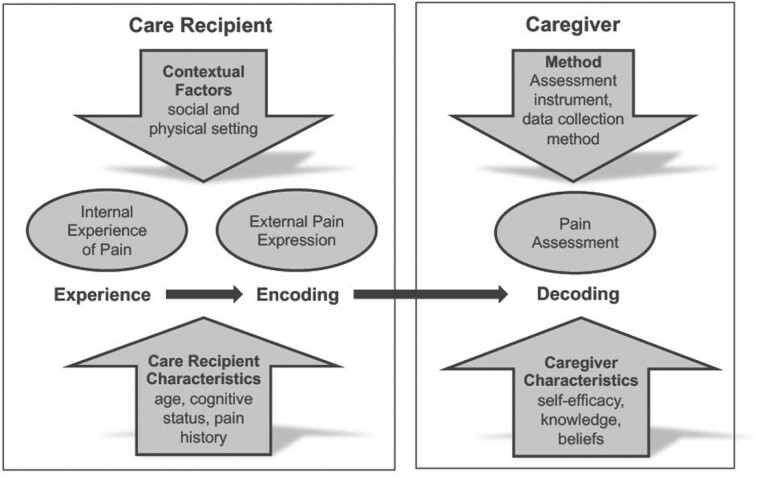
Conceptual framework (adapted from Craig, 2009).

PICT is informed by empirical evidence on behavioral pain assessment and guided by the Communication Model of Pain (Craig, 2015). The model conceptualizes pain communication as a social process in which caregivers play a fundamental role in interpreting their care recipient’s pain behaviors, particularly when self-reported pain is not possible, as in the context of advanced dementia (Gagliese et al., 2018; Hadjistavropoulos & Craig, 2002). The model proposes that pain communication is influenced by characteristics of the care recipient (e.g., level of impairment and pain history), characteristics of the caregiver (e.g., self-efficacy in pain communication, knowledge and beliefs, and prior caregiving experience), contextual factors (e.g., social and physical setting), and method of assessment (e.g., standardized pain assessment tools; Figure 1).

Theories of behavior change (Bandura, 1986; Hekler et al., 2020; Tang & Chan, 2016) corroborate and extend the Communication Model of Pain by highlighting the importance of caregivers’ self-efficacy in promoting accurate pain assessment and communication. Specifically, self-efficacy theory suggests that individuals’ confidence in their ability to perform a specific behavior is a key determinant of whether they will initiate and engage in that behavior. As applied to the PICT intervention, self-efficacy is anticipated to be a core mechanism in motivating caregivers to communicate their care recipient’s symptoms to health care providers.

### PICT Development and Content

Guided by this theoretical framework, a preliminary version of PICT was developed and iteratively refined according to the input of key stakeholders, including caregivers from diverse racial and ethnic backgrounds and health care providers from a range of disciplines, including neurology, primary care, and emergency medicine (Riffin, Patrick, et al., 2022). The final version was manualized in a comprehensive Caregiver Workbook and companion Instructor Manual, which contains directions for conducting each intervention session, descriptions of the training activities, and copies of handouts.

PICT is composed of six modules delivered over four weekly telephone sessions (30–60 min each) by a trained interventionist (Table 1). The first session provides education on the importance of recognizing and treating pain in persons with dementia and addresses widespread myths and misconceptions on this topic. The second and third sessions involve training and practice in identifying pain symptoms using standardized pain assessment tools, including the Iowa Pain Thermometer-Revised (IPT-R; Ware et al., 2015), Pain in Advanced Dementia (PAINAD; Warden et al., 2003), and series of short videos developed by a geropsychologist and a board-certified palliative care physician (Snow et al., 2004). The final session involves coaching in effective strategies for communicating pain symptoms to health care providers. Further materials, including a Pain Diary, Pain Behavior Chart, and Medication List, are provided to participants to facilitate accurate record-keeping and monitoring of their care recipients’ pain over time, as well as effective communication of the identified pain problems to health care providers. Homework exercises in between sessions (10–15 min each) aim to enhance caregivers’ retention of knowledge and promote their self-efficacy through additional practice (e.g., using the PAINAD tool to identify pain symptoms and severity).

**Table 1.** Overview of Modules and Content Covered in the Pain Identification and Communication Toolkit (PICT) Intervention

Session	Modules/topics	Session content
Week 1	1. Overview of PICT manual	• Learning why pain in persons with dementia matters and why it is hard to recognize • Understanding why it’s important to recognize and treat pain • Addressing myths and misconceptions about pain in persons with dementia
	2. Pain in persons with dementia	
Week 2	3. Recognizing pain in dementia	• Understanding why family caregivers are crucial to recognizing and managing pain symptoms • Learning the importance of pain behaviors and how to track them over time • Considering when to use a pain behavior tool and what to do with the information
	4. Using a pain behavior tool	
Week 3	5. The Pain Assessment in Advanced Dementia (PAINAD) tool	• Learning what the PAINAD is and how to use it • Understanding how to interpret PAINAD scores • Learning other behaviors not on the PAINAD • Practicing with the PAINAD while watching video recordings of varying levels of pain • Practicing using a pain behavior chart to record behaviors and track trends over time
Week 4	6. Effective communication with providers	• Coaching in effective communication strategies and establishing a good rapport with providers • Learning how to prepare for medical visits and make the most of the visit time • Practicing ways to share information with providers using a pain diary and medication list

### Study Design

The present study employed a RCT design in which participants completed baseline surveys, were randomized to an intervention (PICT) or control group, and completed postintervention (12-week follow-up) surveys and semistructured exit interviews.

Participants randomized to the intervention condition completed the PICT program with a master’s level social worker who received initial training and ongoing feedback from the Principal Investigator and PICT developer. Initial training involved (a) didactic instruction in how to administer the Instructor Manual and companion Caregiver Workbook, (b) role-playing exercises with study staff to prepare the interventionist to deliver the program to participants, and (c) evaluation of the interventionist’s ability to accurately classify an individual’s pain level and behaviors by comparing her ratings with established metrics. Ongoing support was provided through one-on-one meetings and electronic correspondence with the Principal Investigator and PICT developer, who reviewed difficult cases and responded to specific questions as they arose. Fidelity to the intervention was monitored through biweekly check-ins with the study team.

Participants in the control condition received an informational pamphlet about pain in dementia. The pamphlet described the common causes of pain in persons with dementia, the consequences of untreated pain, strategies for detecting pain, and common treatments. No additional education or follow-up was provided to control group participants.

The study was approved by the Weill Cornell Institutional Review Board (IRB #IRB00009417). All participants provided informed consent and received up to $80 in the form of gift cards, earned for completing each assessment.

### Participants

Recruitment for this study was initiated in March 2019, prior to the onset of the COVID-19 pandemic. During this period, in-person approaches were used to recruit participants from New York City-based medical practices and social service agencies. Medical settings included three academically affiliated outpatient clinics: a geriatrics practice, a memory disorders program, and an internal medicine practice. Social service agencies included senior centers, local Alzheimer’s Association chapters, and area agencies on aging. Caregivers were identified from these settings using a range of strategies, including presentations at health seminars and support groups, mailings to families, flyers posted at each recruitment site, and direct referrals by medical staff, center directors or support group leaders.

With the onset of the COVID-19 pandemic in March 2020, recruitment transitioned to virtual and online approaches. Information about the study was listed on several online outlets, including social media platforms (e.g., facebook.com), online bulletin boards (e.g., patch.com), and research registries (e.g., clinicaltrials.gov and researchmatch.org).

Interested individuals were screened by telephone. Participants were eligible if they were English-speaking, cared for a family member or friend with ADRD and pain, were older than 21, provided care for 8 hr or more a week, lived in the same state as the care recipient, and had internet access. Participants were excluded if they had cognitive impairment (as defined by a score of 10 or less on the Blessed-Orientation-Memory Concentration Test [BOMC]; Katzman et al., 1983), provided care to their care recipient for less than 6 months or for fewer than 8 hr per week, or if their care recipient was enrolled in hospice.

### Measures

Quantitative and qualitative data were collected to evaluate PICT’s feasibility and acceptability, its specific components, and potential to improve caregivers’ confidence in recognizing and communicating about their care recipients’ pain. These data, described in detail below, included (1) recruitment, enrollment, and retention rates tracked throughout the duration of the study, (2) caregiver and care recipient sociodemographic characteristics collected at baseline, (3) caregivers’ self-efficacy in pain-related communication collected at baseline and 12 weeks from all participants, (4) quantitative measures of PICT’s acceptability collected at 12 weeks from caregivers in the intervention condition, and (5) semistructured exit interviews conducted at 12 weeks with caregivers in the intervention condition.

#### Quantitative data

##### Sociodemographic characteristics

Caregiver characteristics included age, gender, ethnicity/race, relationship to person with dementia, living arrangement, marital status, employment status, and educational attainment. Care recipient characteristics included age, gender, and ethnicity/race.

##### Time burden and comprehension

Participants in the intervention group reported (a) whether the amount of information delivered by PICT was acceptable (1 = not enough; 2 = just the right amount; 3 = too much), (b) whether the number of intervention sessions was acceptable (1 = too few; 2 = just the right amount; 3 = too many), and (c) how difficult it was to understand the content of the PICT workbook (1 = very difficult to 5 = not at all difficult).

##### Caregivers’ perceptions about PICT’s potential to improve pain recognition and communication

Intervention group participants were asked for their perceptions regarding PICT’s potential utility, in terms of helping them to (a) identify pain symptoms and (b) communicate pain-related concerns to their care recipient’s health care providers. Response choices on both questions ranged from 1 = not at all to 5 = very.

##### Caregivers’ self-efficacy in pain-related communication

All participants completed the Perceived Efficacy in Patient-Provider Interactions (PEPPI-5; Maly et al., 1998) at baseline and 12-week follow-up. PEPPI-5 is a five-item validated instrument that assesses individuals’ perceived confidence when communicating with health care providers. Questions include, for example, “How confident are you in your ability to know what questions to ask healthcare providers?” All items are answered on a 5-point Likert scale ranging from 1 = “not at all confident” to 5 = “very confident.” The range of possible scores is 5–25, with higher scores indicating greater self-efficacy.

##### Specific components

To inform future adaptations of PICT, intervention group participants were asked how likely they would be to continue using the intervention materials in the future (1 = not at all likely to 5 = extremely likely), including the PAINAD tool, Pain Behavior Chart, Pain Diary, and IPT-R.

#### Qualitative data

##### Exit interviews

Semistructured, in-depth interviews asked intervention group participants to elaborate on their perspectives of PICT and clarify their responses to closed-ended questions (described earlier). The interview guide began with an open-ended question to elucidate participants’ general impressions of intervention, including its overall utility, time burden, and complexity. Follow-up probes asked participants to clarify which of PICT’s components (e.g., PAINAD and IPT-R) were most (and least) helpful in improving their abilities to identify and communicate about pain. Intervention participants were then asked to recount a recent conversation with health care provider(s) about their care recipient’s pain. As part of this exercise, participants were asked to reflect on the conversation and describe whether and how they used PICT materials or strategies to inform the discussion(s). A final question asked participants for any final comments about their participation in the intervention. All interviews were audio-recorded, transcribed, and de-identified.

### Data Analysis

#### Quantitative analyses

Feasibility was assessed by rates of recruitment, enrollment, and retention. Descriptive statistics (means for continuous variables; counts and percentages for categorical variables) were calculated for caregiver and care recipient characteristics at baseline and to evaluate caregivers’ perceptions of PICT’s acceptability. *t* Tests were used to compare between-group differences in caregiver and care recipient characteristics and within-group differences in caregiver self-efficacy in pain-related communication. All analyses were performed in SAS version 9.4 (SAS Institute Inc.).

#### Qualitative analyses

Exit interviews were analyzed through a cyclical, iterative process (Curry et al., 2006), following standard procedures of conventional content analysis (Hsieh & Shannon, 2005). Transcripts were initially reviewed to gain preliminary impressions of the data. Open coding was then used to tag distinct portions of text representing key concepts (Elo & Kyngäs, 2008). Initial codes were collapsed into broader categories by nesting codes within subcategories. Categories were then collapsed into overarching themes to generate a preliminary code structure, which was iteratively refined over the course of data analysis. Coding decisions were made by consensus. To ensure trustworthiness and validity, an audit trail of digital and hard copy data was compiled, cataloguing key analytic decisions and patterns in the data. Microsoft Excel (Microsoft Corporation, 2018) was used to facilitate data organization, management, and analysis (Bree et al., 2016; Ose, 2016).

## Results

### Study Sample Characteristics

**Figure 2.**
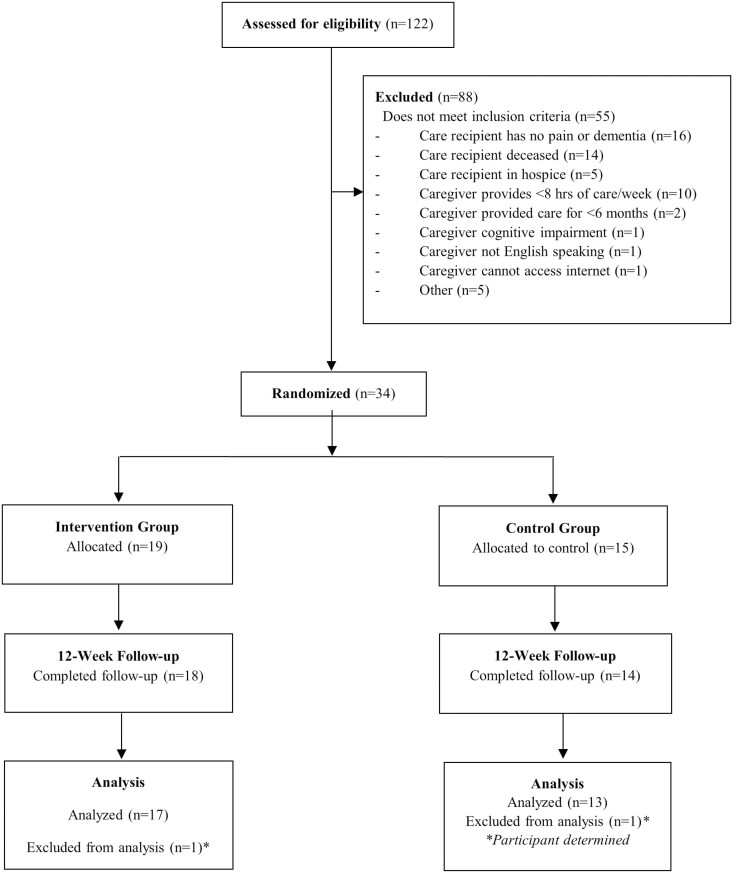
Pain Identification and Communication Toolkit (PICT) pilot test flow diagram.

Enrollment and retention results are presented in Figure 2. A total of 122 individuals were assessed for eligibility. Of these, 55 did not meet the eligibility criteria and 33 were unresponsive to follow-up. The remaining 34 consented to participate and were randomized to PICT (*n* = 19) or the control condition (*n* = 15). All participants randomized to PICT (*n* = 19; 100%) completed the intervention (all four weekly sessions). Most PICT participants (*n* = 18; 94.7%) and control group participants (*n* = 14; 93.3%) completed the 12-week follow-up survey.

No statistically significant between-group differences were observed in caregiver or care recipient characteristics at baseline (Table 2). Caregivers had an average age of 62.7 years. Most caregivers were women (91.2%) and adult children (60.0%) of their care recipient. Two-thirds (68.6%) were non-Hispanic White individuals; 14% were Black and 11% were Hispanic. Nearly half (45.7%) were married. Participants’ care recipients had an average age of 83 years, two-thirds (61.8%) were women, and three-quarters (73.5%) were White.

**Table 2.** Participant Characteristics

Caregiver characteristics	All (*N* = 30)	PICT (*n* = 17)	Control (*n* = 13)	*p* Value
Age, *M* (*SD*)^a^	62.7 (9.9)	63.1 (10.4)	62.2 (9.6)	.80
Female, *n* (%)^a^	27 (90.0%)	14 (82.4%)	13 (100.0%)	.09
Race, *n* (%)^b^				.96
White	22 (73.3%)	13 (76.5%)	9 (69.2%)	
Black	3 (10.0%)	1 (5.88%)	2 (15.4%)	
Other	5 (16.7%)	3 (17.6%)	2 (15.4%)	
Ethnicity, *n* (%)^b^				1.00
Non-Hispanic	27 (90.0%)	15 (88.2%)	12 (92.3%)	
Hispanic	3 (10.0%)	2 (11.8%)	1 (7.7%)	
Marital status, *n* (%)^b^				.86
Married	13 (43.3%)	9 (52.9%)	4 (30.8%)	
Single	8 (26.7%)	4 (23.5%)	4 (30.8%)	
Divorced	8 (26.7%)	4 (23.5%)	4 (30.8%)	
Widowed	1 (3.3%)	0 (0.00%)	1 (7.7%)	
Employment, *n* (%)^b^				.66
Working full time	4 (13.3%)	2 (11.8%)	2 (15.4%)	
Working part time	5 (16.7%)	2 (11.8%)	3 (23.1%)	
Retired	12 (40.0%)	8 (47.1%)	4 (30.8%)	
Other	9 (26.7%)	5 (29.4%)	4 (30.8%)	
Education level, *n* (%)^c^				.99
Advanced degree	7 (23.3%)	3 (17.6%)	4 (30.8%)	
Some graduate school	4 (13.3%)	3 (17.6%)	1 (7.7%)	
College graduate	15 (50.0%)	9 (52.9%)	6 (46.2%)	
Other	4 (13.3%)	2 (11.8%)	2 (15.4%)	
Relationship to person with dementia, *n* (%)^c^				
Spouse	9 (26.7%)	6 (35.3%)	3 (23.1%)	.92
Child	19 (63.3%)	11 (64.7%)	8 (61.5%)	
Other	3 (10.0%)	1 (5.9%)	2 (15.4%)	
Lives with person with dementia, *n* (%)^a^	24 (80.0%)	14 (82.4%)	10 (76.9%)	
Care recipient characteristics				
Age, *M* (*SD*)^a^	83.0 (8.9)	82.5 (9.7)	83.7 (8.0)	.70
Female care recipient, *n* (%)^a^	19 (63.3%)	12 (70.6%)	7 (53.8%)	.38
Ethnicity, *n* (%)^b^				.48
Non-Hispanic	25 (83.3%)	15 (88.2%)	10 (76.9%)	
Hispanic	2 (6.7%)	1 (5.9%)	1 (7.7%)	
Unknown	3 (10.0%)	1 (5.9%)	2 (15.4%)	
Race, *n* (%)^b^				1.00
White	22 (73.3%)	14 (82.4%)	8 (61.5%)	
Black	4 (13.3%)	1 (5.9%)	3 (23.1%)	
Other	3 (10.0%)	2 (11.8%)	2 (15.4%)	

*Notes*: *M* = mean; PICT = Pain Identification and Communication Toolkit; *SD* = standard deviation.

Other caregiver race: *n* = 3 multiracial, *n* = 2 Asian; Other patient race: *n* = 2 Asian, *n* = 1 multiracial.

^a^Significance determined by independent sample *t* tests.

^b^Significance determined by Fisher exact test.

^c^Significance determined by one-way ANOVA.

Results from our quantitative and qualitative data collected from PICT group participants are organized into five sections, presented below, that reflect participants’ (1) general impressions of PICT, (2) perceptions of PICT’s time burden and comprehension, (3) views regarding PICT’s potential to improve pain recognition and communication, (4) perspectives on the utility of PICT’s specific components, and (5) recommendations for future refinement and implementation.

### General Impressions of PICT

Overall, participants shared positive perspectives of the intervention, with the majority (83.4%) reporting that they would be likely or very likely to recommend PICT to other caregivers. One participant explained: “PICT helps to tell the whole picture of what’s going on [with care recipient’s pain] … It helps facilitate the doctor’s appointments to the best that they can possibly be.” Others clarified that “having clear-cut examples and specifications really helped everything [pain-related] make more sense” and “made my observations [of care recipient’s pain] better.”

Caregivers with varying degrees of prior experience with pain and dementia articulated benefits of the program. Caregivers with limited prior experience reported that PICT “was helpful because of the in-depth knowledge that I’ve acquired since joining the program.” One participant elaborated: “It was really good for [PICT] to be initiated early on … I made several copies [of the handouts] so I had them ready to go. Now, I’m all of a sudden in this major decline … I already had the information ready, so it’s much easier for me to just implement those strategies.” Caregivers with greater prior experience felt that PICT provided them with important reminders that helped to reinforce their existing knowledge and skills:

PICT gave me a heightened awareness of the necessity for clear communication. There’s just some language in the toolkit that, even if it’s not all new to me, it’s good to be reminded… When things are the most stressful, it’s easiest to forget what you know. That’s when having something physical to go to and look it up and say, ‘oh I should do this’ can be very helpful.

### Time Burden and Comprehension

Virtually all participants (94.1%) reported that one intervention session per week was acceptable, and three-quarters (77.8%) reported that the PICT workbook had just the right amount of information: “PICT is a concise tool … It’s efficient. It’s an efficient use of your time and an efficient use of the physician’s.” Participants who reported that the length and content were too short explained: “I hated for [PICT] to end because it was so helpful. And I wish that it went a little longer.” The majority of participants (89.5%) thought that PICT’s content was easy or very easy to understand. As one caregiver commented, “The behaviors and the definitions most definitely were very clear. [They] were not difficult to understand at all.” Another reiterated that “the written materials are easy to read. I think they would be accessible to know, lots of different people.”

### Potential to Improve Pain Recognition

Two-thirds (66.7%) of participants felt that PICT helped them to recognize their care recipient’s pain symptoms. Caregivers remarked that after completing PICT they were “much better able to identify pain and [use] … non-pharmacological solutions to [address] the pain.” They reported gaining knowledge about “pain symptoms that I was not aware was a [pain] symptom” and “a better understanding of what symptoms are correlated with pain and what degree they might indicate.” One caregiver described PICT as “extremely helpful because … [symptoms] that I never thought that were part of this [pain] behavior, I just assumed was part of getting old.” Another participant elaborated on this point by describing how she used PICT’s content to inform behavioral assessment and management of her care recipient’s pain:

[Care recipient] would tell me he was an eight or a nine [on a 10-point pain scale], but his facial expression did not indicate to me an eight or a nine. I was able to identify other signs that could give me a clue of where he was at with his pain. He’s done a lot more grimacing and moaning… So, I am now better able to identify the pain, and then to take actions [for example] to give him a massage, change positions - some of those non-medical, non-pharmacological solutions to reduce the pain.

The various modes of instruction (didactic content of the workbook, videos of pain expression, and role-playing with the interventionist) were viewed as a strength of the program, affording caregivers with various opportunities to practice identifying pain symptoms. One participant explained:

Not just reading [about pain in dementia], but actually by doing something. So, we have the visual, the auditory, the actual performances, the doing. By using all of the teaching modalities, ways that people learn. I think that’s what made the book have, or give me, anyway, all of that confidence.

### Potential to Improve Pain Communication

Most participants (83.3%) felt that PICT was a useful tool in helping them to communicate about their care recipient’s pain with health care providers. Indeed, caregivers who were in the PICT group showed a significant positive change from baseline (*M* = 19.84) to 12 weeks (*M* = 21.77) in self-efficacy in communicating with health care providers (T2−T1: *M* = 1.93, *p* = .02); caregivers in the control group did not show a significant change (T2−T1: *M* = 0.18, *p* = .95; Table 3).

**Table 3.** Caregiver Self-Efficacy in Pain-Related Communication

Group assignment	Baseline (T1)	12 Weeks (T2)	T1−T2	*p* Value
	*M* ± *SD*	*M* ± *SD*		
PICT	19.84 ± 4.40	21.89 ± 2.30	2.05	.02
Control	21.73 ± 2.81	21.71 ± 3.05	−0.02	.95

*Note*: PICT = Pain Identification and Communication Toolkit.

One caregiver explained, “I think our communication is improved because I can actually give [the health care team] a very thorough description of the pain and the progression of the pain, which helps them in deciding what [the] treatment options are.” Participants further described how they used the terminology they learned through the PICT program to enhance pain-related discussions with medical professionals: “[PICT] helped me to communicate with the nurses because I was using this specific terminology to say [care recipient’s] body is rigid, or she has facial grimacing, or occasional labored breathing. I was using these terms to communicate [and] report [the symptoms].” Another confirmed: “I had more confidence in that I was understanding what was going on with my mom … because I had the terminology.”

Beyond gaining a new vocabulary related to pain symptoms, caregivers reported that PICT helped to validate their role as an advocate for their care recipient: “[What was] helpful was just the reinforcement [that at] appointments it is appropriate, and even necessary, to be an advocate and how to be an advocate. Just having that messaging is reinforcing.” Another reiterated: “[Providers] need to start seeing caregivers in a different light. PICT certainly gives you the tools that give confidence to start asking the right questions and showing that you’re the advocate and establishing that relationship with providers.”

### Utility of Specific Components

With respect to PICT’s components, nearly three-quarters of participants reported that they would be likely or very likely to continue using the PAINAD tool (72.2%) and Pain Behavior Chart (72.2%) in the future. One caregiver clarified: “The PAINAD tool was very effective for me. Taking [care recipient’s] verbalizations … what she’s saying and really correlating them with her body language was helpful.” Another described the benefits of the Pain Behavior Chart: “I realized that there’s a pattern … I graphed it with a pen or a pencil. I made photocopies so every time we went [to a healthcare appointment] I used that chart.” Another emphasized: “[The Pain Behavior Chart] put me in a different frame of mind. As if I came with business today … Made me more confident or the most prepared to discuss things with the providers.” Overall, caregivers felt that these tools played an important role in health care visits. One caregiver remarked, “It helps me not to forget anything when I had to talk to the doctors. It made it clear for them to understand.”

Two-thirds of intervention participants stated that they would be likely to use the Pain Diary (66.7%) in the future and half (50%) reported that they would use the IPT-R. One participant commented that the Pain Diary helped her to “see a trend at different times [during the] day. I know [care recipient] always gets up with pain and that’s where I could see that. Just getting up. The up-and-down is what triggers his pain.” Another reported that using the Pain Diary “made me realize that there [are] more behavior changes … I wouldn’t have realized that unless I noted it because of these charts and these graphs.” However, this tool was not viewed as uniformly helpful: “I’m not sure I felt I could put enough on the Pain Diary … to assist [health care providers] … If I was going to the right specialists, they could care, but [care recipient’s] primary [care doctor] … wants to be the one to find [the pain problem] or acknowledge it.” With respect to the IPT-R, one caregiver noted that although “it was a very good tool … for me it wasn’t as helpful because we already established [patient’s pain] on just a 0 to 10 scale.” Another corroborated that the tool was a bit redundant “because we were used to a different system … using a plain score of 0 to 10.”

### Recommendations for Future Refinement and Implementation

Participants’ recommendations for advancing PICT centered on two key areas: (1) technology-based adaptations of the PICT workbook and (2) integration of PICT within systems of care delivery.

With respect to technology-based adaptations, participants commented that “the only thing that I would change is to have [PICT] available in a digital format as well.” They asserted that “some [caregivers] might benefit from an electronic version of some [handouts],” further suggesting that “it would be interesting to have a version of the workbook where you can [get an email] link right away and get integrated [information] about pain assessment.” Another participant recommended “having [PICT] in an app.” One caregiver elaborated: “So many people, you know, their phones are with them all the time … [A PICT app] would be a very easy thing for someone to use as they’re caring for someone. You have all the information with you when you’re going to an appointment.”

With respect to PICT’s potential for integration within systems of care delivery, caregivers commented that the program “would be a great program for health care systems” or “for a facility or assisted living community.” One caregiver commented that “the whole PICT workbook was a really positive experience. I would think every … assisted living caregiver and worker should have these and be a part of their training as well.” Several caregivers recounted prior interactions in which health care providers inquired about accessing the PICT tools, further highlighting the need to embed PICT’s elements within clinical practice: “When I brought [the PICT materials] to the doctors, they said, ‘We need to have this at our hospital. Where did you get this?’” Beyond traditional health care settings, participants asserted that PICT would be useful for in-home care delivery. One caregiver thought it would be useful to “teach [PICT] to their home attendant so they could know … They have no training … I was able to take the knowledge that I know … and was able to teach that to [care recipient’s] home attendants.”

## Discussion and Implications

This study reports findings from an initial evaluation of the PICT, a novel intervention to help family caregivers of persons with dementia to recognize and communicate about their care recipient’s pain symptoms. Findings indicate that PICT is a promising intervention with high feasibility, as demonstrated by the strong retention rate for the intervention sessions (i.e., 100% completion), limited loss to follow-up at 12 weeks, and positive caregiver appraisals of the time burden (i.e., reported satisfaction with the frequency of intervention sessions and amount of information provided). PICT’s utility and potential to improve caregivers’ pain recognition and communication are further evidenced by caregivers’ favorable appraisals of PICT’s content and specific components, including structured pain assessment (e.g., PAINAD) and monitoring tools (e.g., Pain Behavior Chart). Results also suggest potential opportunities for further development and enhancement of PICT, including technology-based adaptations and possible integration within care delivery systems.

PICT extends prior research on pain assessment in persons with dementia by translating relevant findings from observational studies into a practical intervention that is poised for future efficacy testing, and if successful, implementation and evaluation in real-world practice contexts. Laboratory-based studies in highly controlled settings have demonstrated that standardized pain assessment instruments (e.g., PAINAD) can significantly improve caregivers’ accuracy in detecting pain in persons with dementia (Ammaturo et al., 2017; Bullock et al., 2019; Porter et al., 2022). Meanwhile, qualitative research has indicated that written records (e.g., pain logs and behavior charts) and collaborative approaches to pain management may facilitate pain-related communication among family caregivers and health care providers (Riffin, Patrick, et al., 2022). To our knowledge, PICT is the first intervention to integrate these elements into a multicomponent intervention for use by family caregivers of persons with dementia that provides training in evidence-based pain assessment and communication techniques.

Following the NIH Stage Model of intervention development, this Stage I pilot trial provides foundational evidence to merit further evaluation in a fully powered Stage II trial designed to rigorously test PICT’s efficacy and ascertain its mechanism(s) of action. Theories of pain communication and behavior change suggest two mechanisms by which PICT may operate: caregivers’ self-efficacy and pain-related knowledge. Self-efficacy is a modifiable attribute that can be reinforced by coaching and feedback to reinforce the initial skills training. PICT extends this premise by providing caregivers with one-on-one coaching by a trained interventionist as well as regular opportunities for practice. Caregiver knowledge—including understanding of and beliefs about pain—has also been implicated as an important predictor of accurate pain identification (Knopp-Sihota et al., 2019). Evaluation of these putative mechanisms of action will not only help to guide subsequent phases of our own research but also provide valuable data to other researchers regarding the key drivers and malleable processes to be targeted in future research.

Participants’ recommendations for technology-based adaptations of PICT and future integration within systems of care delivery align with priorities identified by national initiatives (RAISE Family Caregiver Council, 2021; Riffin, Griffin, et al., 2022) and with recent reviews underscoring the need for broader translation of caregiver interventions into practice (Dilworth-Anderson et al., 2020; Hodgson & Gitlin, 2021). Population-based data indicate that fewer than 1 in 10 family caregivers in the United States receive role-related training (Burgdorf et al., 2019), further reflecting the limited uptake of caregiver interventions in real-world settings.

Implementation science frameworks will be critical to understanding how caregiver interventions, including PICT, can be embedded within care delivery contexts, with specific consideration given to the tension between fidelity (adherence to the original version of PICT) and adaptation (including technology enhancements and modifications to a within local practice). Further, although technology has been widely embraced as a strategy to promote broader diffusion of evidence-based programs, particularly for rural and long-distance caregivers (Wang et al., 2021), future work will need to address the “digital divide” whereby individuals from traditionally marginalized communities (i.e., those with lower socioeconomic statuses; racial and ethnic minorities) experience barriers to accessing and using technology (Anderson & Kumar, 2019).

This research is subject to several limitations. First, as a pilot study, this trial was not sufficiently powered to evaluate between-group differences in pain recognition or communication. A full-scale efficacy trial will be necessary to evaluate changes in these outcomes over time and across treatment groups. To verify caregivers’ accuracy in pain recognition, future work will need to incorporate independent, external assessments; for example, by comparing caregiver ratings on a standardized assessment tool (e.g., the PAINAD) with ratings of an independent evaluator (e.g., an expert or professional). Second, despite using a range of recruitment strategies, encompassing direct (targeted letters and phone calls) and indirect (advertisements via flyers, social media, and online bulletin boards) approaches, the generalizability of our study findings may be limited. Further, although caregivers were recruited from across the United States and represented diverse racial and ethnic backgrounds, the majority were well-educated and from the New York area; thus, replication and expansion of the study sample are needed to confirm PICT’s potential benefits. Third, for the purposes of this proof-of-concept study, our control condition was a simple pamphlet on pain and dementia. Future work will need to compare the efficacy of PICT with an attention control condition that is analogous to PICT in terms of time commitment and mode of delivery and includes comparable activities to control for elements such as social engagement, experiential learning, and therapeutic alliance. Finally, we did not have access to the care recipients’ medical records and could not confirm pain and dementia diagnoses. Further, although we collected caregivers’ reports of their care recipient’s general health status, we did not ascertain dementia severity or duration. Future research would benefit from evaluating PICT in the context of health or long-term care settings where care recipients’ data (e.g., sociodemographic characteristics, diagnoses, pain treatments, and health service use, including hospitalization or institutionalization) can be abstracted from the medical record.

## Conclusion

Caregiver training in pain assessment and subsequent communication of these results to a health care provider has the potential to help address underdetection and undermanagement of pain in persons with dementia. This study represents a critical step in addressing these issues by conducting a preliminary evaluation of PICT, a theory-driven, multicomponent intervention designed to train caregivers of persons with dementia in pain symptom recognition and communication. Findings from this study provide initial evidence of PICT’s promise as a feasible intervention that is highly acceptable to caregivers and poised for future efficacy testing, adaptation, and scaling.
